# Supplementary material for: Chronic stress disrupts the network among stress granules, P-bodies, and motor proteins
Source: bioRxiv. 2026 Jan 11:2026.01.09.698697. Preprint. [Version 1] doi: 10.64898/2026.01.09.698697 (PMC12803230; doi:10.64898/2026.01.09.698697)
Supplement: Supplement 2 [file media-2.pdf]

Table S1. Cell lines used in this study and their sources.

Cell line	Source
U2OS-WT	ATCC
U2OS-G3BP1/G3BP2 KO double (DDG3BP1/2)	(Kedersha et al., 2016)
U2OS-GFP-G3BP1/G3BP2 double KO (GFP-DDG3BP1/2)	(Kedersha et al., 2016)
U2OS-GFP-G3BP1-G3BP1/G3BP2 double KO (GFP-G3BP1-DDG3BP1/2)	(Kedersha et al., 2016)
U2OS-UBAP2L KO (DUBAP2L)	(Sanders et al., 2020)
U2OS-G3BP1 KO (DG3BP1)	(Kedersha et al., 2016)
U2OS-G3BP2 KO (DG3BP2)	(Kedersha et al., 2016)
MDA-MB-468-WT	ATCC
MDA-MB-468-G3BP1/G3BP2 KO double (DDG3BP1/2)	(Yang et al., 2024)

Table S2. Details of siRNAs used in this study.

Target	siRNA	Source
MYH9 (Human)<br>Entrez Gene 4627	siGENOME Human MYH9 siRNA – SMARTpool, targeting:<br><br>GUAUCAAUGUGACCGAUUU<br><br>CAAAGGAGCCCUGGCGUUA<br><br>GGAGGAACGCCGAGCAGUA<br><br>CGAAGCGGGUGAAAGCAAA	Dharmacon (A Horizon Discovery Group Company) Discovery, cat no. L-007668-00
Non-Targeting control	siGENOME Non-Targeting siRNA Pool #2, targeting:<br><br>UAAGGCUAUGAAGAGAUAC<br><br>AUGUAUUGGCCUGUAUUAG<br><br>AUGAACGUGAAUUGCUCAA<br><br>UGGUUUACAUGUCGACUAA	Horizon Discovery, cat no. D-001206-14

Table S3. Information for antibodies used in the study.

Antigen	Host species	Catalog number	Source	IF dilution	WB dilution
G3BP1	Mouse	sc-365338	Santa Cruz Biotechnology, Inc.	1:200	1:1000
eIF3b	Rabbit	PA5-117928	Invitrogen	1:200	N/A
MYH9	Mouse	60233-1-Ig	Protein tech	N/A	1:1000
b-actin	Mouse	66009-1-Ig	Protein tech	N/A	1:2000
G3BP1	Rabbit	A302-034A	Bethyl Labs	1:200	N/A
HEDLS/ P70 S6 kinase	Mouse	sc-8418	Santa Cruz Biotechnology, Inc.	1:200	N/A
UBAP2L	Rabbit	A300-534A	Bethyl Labs	N/A	1:1000
DDX6	Rabbit	A300-461A	Bethyl Labs	N/A	1:1000
EDC3	Mouse	sc-365024	Santa Cruz Biotechnology, Inc.	N/A	1:1000
G3BP2	Rabbit	A302-040A	Bethyl Labs	N/A	1:1000
USP10	Mouse	sc-365828	Santa Cruz Biotechnology, Inc.	N/A	1:500
Caprin1	Rabbit	15112-1-AP	Protein tech	N/A	1:1000
G3BP1	Mouse	sc-365338	Santa Cruz Biotechnology, Inc.	1:500	1:1000
p-eIF2a		sc-8418	Santa Cruz Biotechnology, Inc.	1:200	N/A
Total eIF2a		10970	Protein Tech	1:200	N/A
puromycin		sc-1751	Santa Cruz Biotechnology, Inc.	1:500	N/A
Cy3 AffiniPure™ Goat Anti-Rabbit IgG (H + L)		111-165-003	Jackson Immuno	1:2000	
Cy™5 AffiniPure Goat Anti-Mouse IgG (H + L)		115-005-003	Jackson ImmunoResearch	1:200	
Cy™2 AffiniPure Goat Anti-Rabbit IgG (H + L)		111-005-003	Jackson ImmunoResearch	1:200	
Alexa Fluor® 488 AffiniPure® Goat Anti-Mouse IgG, F(ab')$_2$ fragment specific		115-545-006	Jackson ImmunoResearch	1:200	
Peroxidase-Conjugated AffiniPure Donkey anti-Mouse	Donkey	715-035-150	Jackson ImmunoResearch	NA	1:5000
Peroxidase-Conjugated AffiniPure Donkey anti-Goat) at 1:5000	Donkey	711-005-152	Jackson ImmunoResearch	NA	1:5000

Supplementary Figure 1.

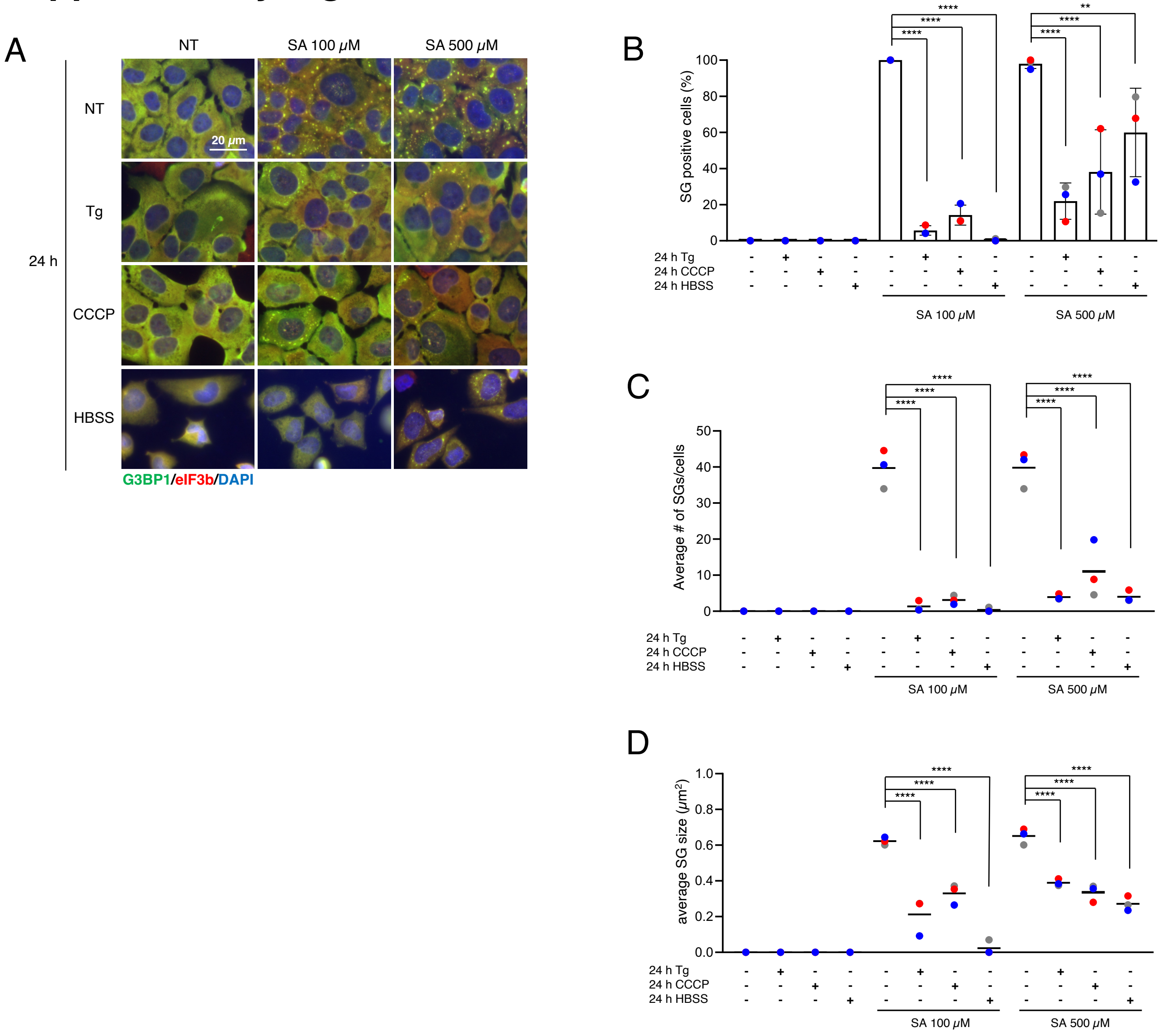

Supplementary Figure 1. Chronic incubation of Tg, CCCP, and HBSS also decreases the formation and size of SG. (A–D) U2OS cells were subjected to treatment with 100 or 500 μM SA for 1 h after preincubation with 1 μM Tg, 60 μM CCCP or HBSS for 24 h. Unstressed cells (NT) were used as a control. (A) Representative images of U2OS cells stained with G3BP1 (green), eIF3B (red), and DAPI (blue) after the cells had been subjected to specific stresses. Scale bar = 20 μm. (B) Cells were examined for the presence of the core SG markers G3BP1, and eIF3B. (C) Cells were examined for the average number of G3BP1 per cell. (D) Cells were examined for the average size of G3BP1. (B–D) P values were assessed using a one-way ANOVA ($p^{**} < 0.01$, $p^{****} < 0.0001$) Results are mean ± S.E.M. (n = 12).

Supplementary Figure 2.

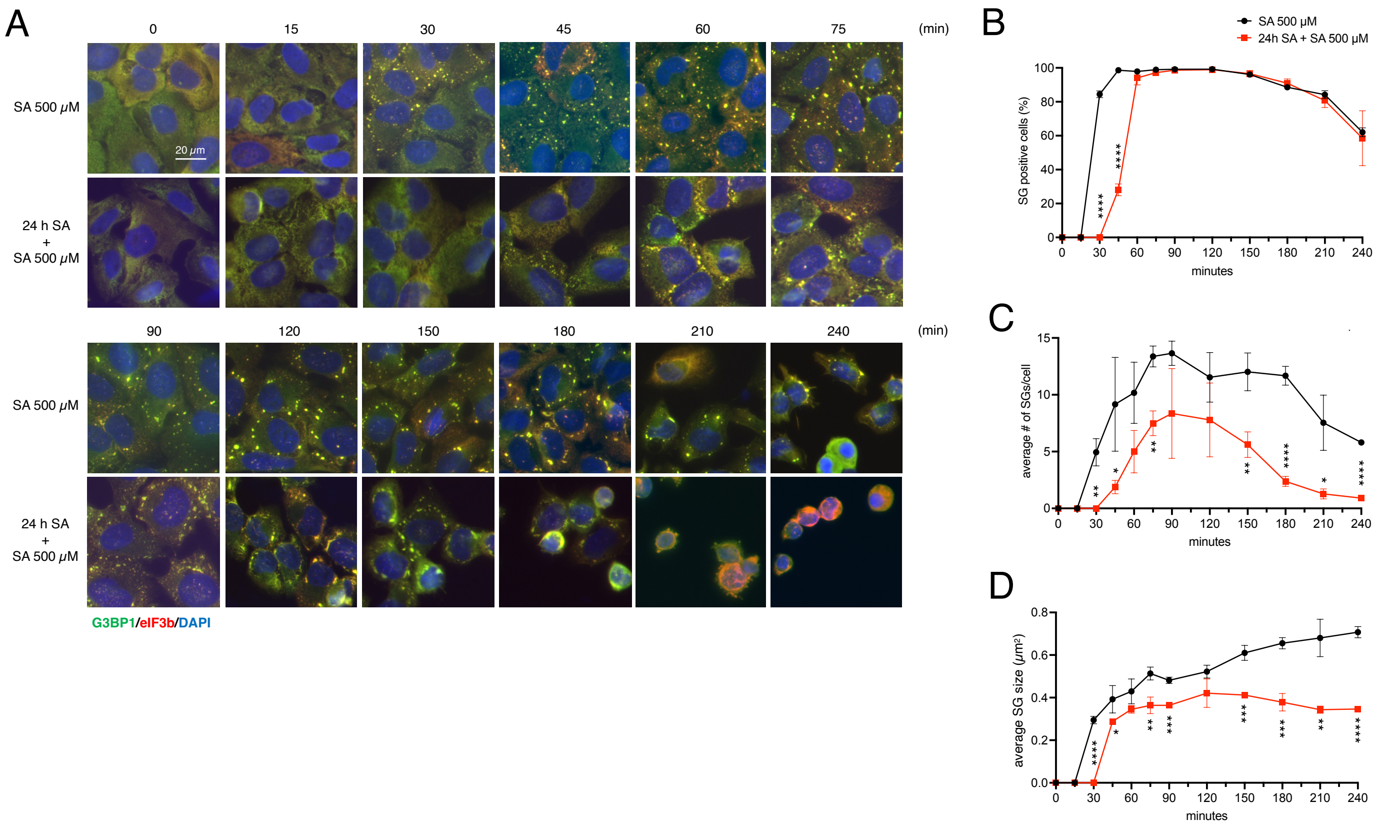

Supplementary Figure 2. Chronic stress negatively regulates the size of SGs at any time points of SG induction

(A–D) U2OS cells were subjected to treatment with 500 μM SA for 15, 30, 45, 60, 75, 90, 120, 150, 180, 210, 240 min after preincubation with 10 μM SA for 24 h. Unstressed cells (NT) were used as a control. (A) Representative images of U2OS cells stained with G3BP1 (green), eIF3B (red), and DAPI (blue). Scale bar = 20 μm. (B) Cells were examined for the presence of the core SG markers G3BP1, and eIF3B. (C) Cells were examined for the average number of G3BP1 per cell. (D) Cells were examined for the average size of G3BP1. (B–D) Four images were taken for each of the three biological replicates (independent experiments). P values were assessed using a one-way ANOVA (p* < 0.05, p** < 0.01, p*** < 0.001, p**** < 0.0001). Results are mean ± S.E.M. (n = 3).

Supplementary Figure 3.

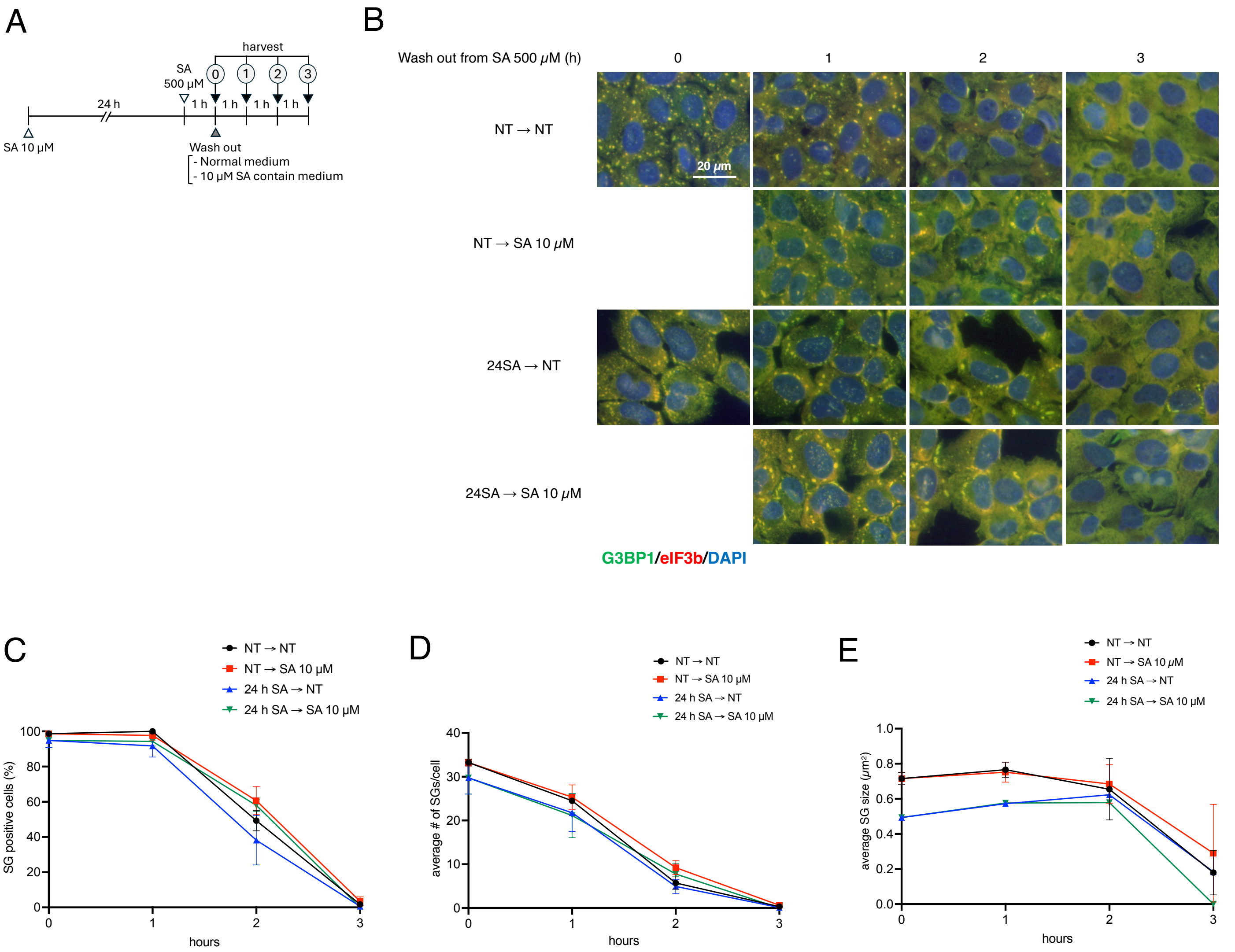

Supplementary Figure 3. Chronic stress does not affect the disassembly of SG

U2OS cells were subjected to treatment with 500 μM SA for 1 h after preincubation with 10 μM SA for 24 h prior to changing the medium to fresh or 10 μM SA contained medium. Unstressed cells (NT) were used as a control. (A) Schematic illustration of the experimental timeline. (B) Representative images of U2OS cells stained with G3BP1 (green), eIF3B (red), and DAPI (blue). (C) Cells were examined for the presence of the core SG markers G3BP1, and eIF3B. Scale bar = 20 μm. (D) Cells were examined for the average number of G3BP1 per cell. (E) Cells were examined for the average size of G3BP1. (C–E) Results are mean ± S.E.M. (n = 3).

Supplementary Figure 4.

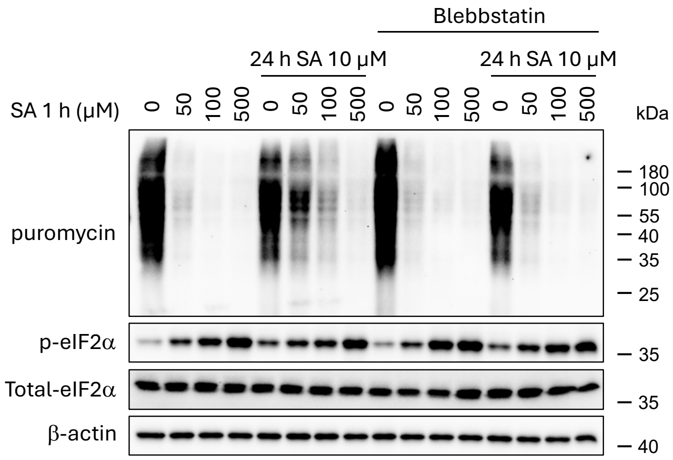

Supplementary Figure 4. Blebbstatin does not rescue translation under chronic stress

U2OS cells were subjected to treatment with 50, 100, or 500 μM SA for 1 h after pre-incubation with 10 μM SA and/or 100 μM of blebbstatin for 24 h. Unstressed cells (NT; SA 0 μM) were used as a control. Cells were pulsed with puromycin and emetine for 5 min and lysed. Cell lysates were subjected to western blotting using antibodies for Puromycin, p-eIF2α, total eIF2α, and β-actin.

Supplementary Figure 5.

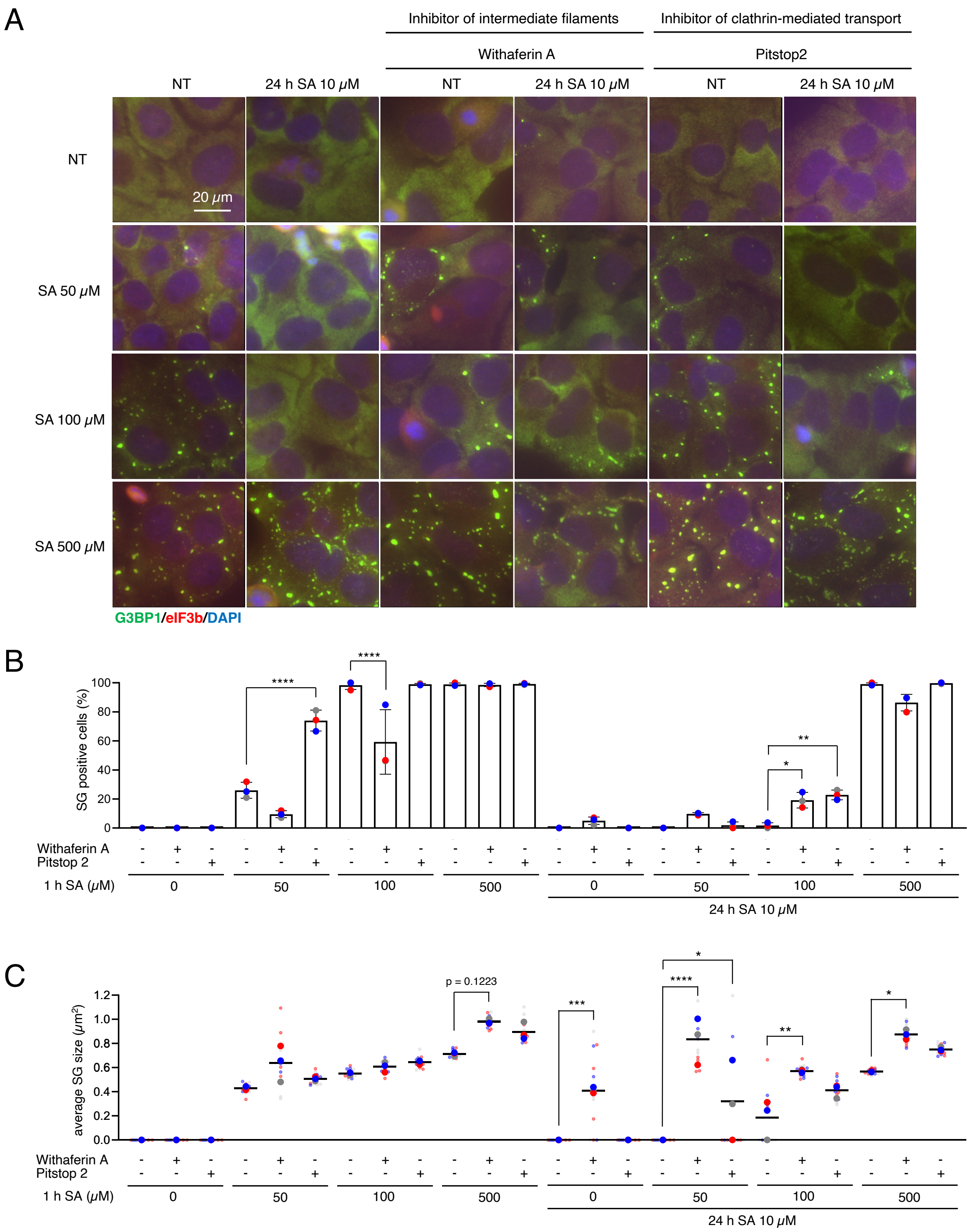

Supplementary Figure 5. Inhibitors of intermediate filaments and Clathrin-mediated transport positively regulate the SGs

(A–C) U2OS cells were subjected to treatment with 50, 100, or 500 μM SA for 1 h after preincubation with 10 μM SA and/or 1 μM withaferin A and 30 μM Pitstop 2 for 24 h. Unstressed cells (NT) were used as a control. (A) Representative images of U2OS cells stained with G3BP1 (green), eIF3B (red), and DAPI (blue). Scale bar = 20 μm. (B) Cells were examined for the presence of the core SG markers G3BP1, and eIF3B. (C) Cells were examined for the average size of G3BP1. (B–C) Four images were taken for each of the three biological replicates (independent experiments). Large circle dots represent the mean results from the images (n = 4 technical replicates) of one biological replicate, the same colors of dots are from the same independent experiment. Small circle dots in different shades of the same color are from the same independent experiment. P values were assessed using a one-way ANOVA ($p^* < 0.05$, $p^{**} < 0.01$, $p^{***} < 0.001$, $p^{****} < 0.0001$). Results are mean ± S.E.M. (n = 3).

Supplementary Figure 6.

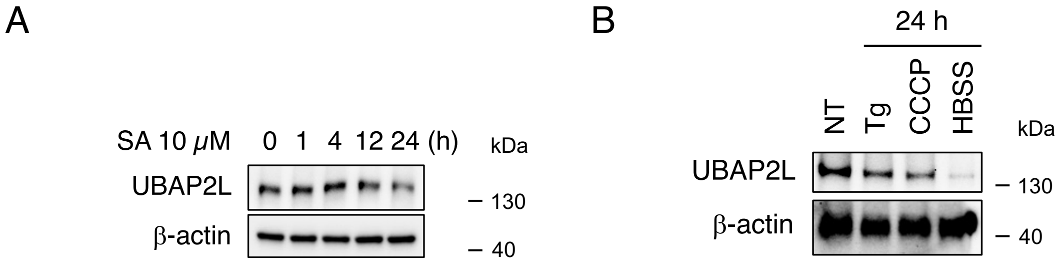

Supplementary Figure 6. Chronic stresses decrease the protein level of UBAP2L

(A) U2OS cells were subjected to treatment with 10 μM SA for 0, 1, 4, 12, and 24 h. (B) U2OS cells were subjected to treatment with 1 μM Tg, 60 μM CCCP, or HBSS for 24 h. (A–B) Cell lysates were subjected to western blotting using antibodies for UBAP2L and β-actin.

Supplementary Figure 7.

A

1	**MAQQAADKYL**	**Y**VDKNFINNP	LAQADWAAKK	LVWVPSDKSG	FEPASLKEEV	50
51	GEEAIVELVE	NGKKVKVNKD	DIQKMNPPKF	SKVEDMAELT	CLNEASVLHN	100
101	LKERYYSGLI	YTYSGLFCVV	INPYKNLPIY	SEEIVEMYKG	KKRHEMPPHI	150
151	YAITDTAYRS	MMQDREDQSI	LCTGESGAGK	TENTKKVIQY	LAYVASS**HKS**	200
201	**KKDQGEL**ERQ	LLQANPILEA	FGNAK**TVKND**	**N**SSRFGKFIR	INFDVNGYIV	250
251	GANIETYLLE	KSRAIRQAKE	ERTFHIFYYL	LSGAGEHLKT	DLLLEPYNKY	300
301	RFLSNGHVTI	PGQQDKDMFQ	ETMEAMRIMG	IPEEEQMGLL	RVISGVLQLG	350
351	NIVF**KKERNT**	**DQASMPDNT**A	AQKVSHLLGI	NVTDFTRGIL	TPRIKVGRDY	400
401	VQKAQTKEQA	DFAIEALAKA	TYERMFRWLV	LRINKALDKT	KRQGASFIGI	450
451	LDIAGFEIFD	LNSFEQLCIN	YTNEKLQQLF	NHTMFILEQE	EYQREGIEWN	500
501	FIDFGLDLQP	CIDLIEKPAG	PPGILALLDE	ECWFPKATDK	SFVEKVMQE**Q**	550
551	**GTHPKFQKPK**	**QLKD**KADFCI	IHYAGKVDYK	ADEWLMKNMD	PLNDNIATLL	600
601	HQSSDKFVSE	LWKDVDR**IIG**	**LDQVAGMSET**	**ALPGAFKTRK**	**GMFR**TVGQLY	650
651	KEQLAKLMAT	LRNTNPNFVR	CIIPNHEKKA	GKLDPHLVLD	QLRCNGVLEG	700
701	IRICRQGFPN	RVVFQEFRQR	YEILTPNSIP	KGFMDGKQAC	VLMIKALELD	750
751	SNLYRIGQSK	VFFRAGVLAH	LEEERDLKIT	DVIIGFQACC	RGYLARKAFA	800
801	KRQQQLTAMK	VLQRNCAAYL	KLRNWQWWRL	FTKVKPLLQV	SRQEEEMMAK	850
851	EEE**LVKVREK**	**QLA**AENRLTE	METLQSQLMA	EKLQLQEQ**LQ**	**AE**TELCAEAE	900
901	ELRARLTAKK	QELEEICHDL	**EARVEEEEER**	**CQHLQAEKKK**	**MQQNIQ**ELEE	950
951	**QLEEEESARQ**	**KLQLEKVTTE**	**AKLKKLEEEQ**	**IILEDQNCKL**	**AKEKKLLEDR**	1000
1001	**IAEFTTNLTE**	**EEEKSKSLAK**	**LKNKHEAMIT**	**DLEERLRREE**	**KQRQELEKTR**	1050
1051	**RKLEGDSTDL**	**SDQIAELQAQ**	**IAELKMQLAK**	**KEEELQAALA**	**RVEEEAAQKN**	1100
1101	**MALKKIRELE**	**SQISELQEDL**	**ESERASRNKA**	**EKQKRDLGEE**	**LEALKTELED**	1150
1151	**TLDSTAAQQE**	**LRSKREQEVN**	**ILKKTLEEEA**	**KTHEAQIQEM**	**RQKHSQAVEE**	1200
1201	**LAEQLEQTKR**	**VKANLEKAKQ**	**TLENERGELA**	**NEVKVLLQGK**	**GDSEHKRK**KV	1250
1251	EAQ**LQELQVK**	**FNEGERVRTE**	**LADKVTKLQV**	**ELDNVTGLLS**	**QSDSKSSKLT**	1300
1301	**K**DFS**ALESQL**	**QDTQELLQEE**	**NRQKLSLSTK**	**LKQVEDEKNS**	**FREQLEEEEE**	1350
1351	**AKHNLEKQIA**	**TLHAQVADMK**	**KKMEDSVGCL**	**ETAEEVKRKL**	**QKDLEGLSQR**	1400
1401	**HEEKVAAYDK**	**LEKTKTRLQQ**	ELDDLLVDLD	HQRQSACNL**E**	**K**KQKKFDQLL	1450
1451	**AEEKTISAKY**	**AEERDRAEAE**	**AREKETKALS**	**LARALEEAME**	**QKAELERLNK**	1500
1501	**QFRTEMEDLM**	**SSKDDVGKSV**	**HEL**EKSKRAL	E**QQVEEMKTQ**	**LEELEDELQA**	1550
1551	**TEDAKLRLEV**	**NLQAMKAQFE**	**RDLQGRDEQS**	**EEKKKQLVRQ**	**VREMEAELED**	1600
1601	**ERKQRSMAV**A	ARKKLEMDLK	DLEAHIDSAN	**KNR**DEAIKQL	RKLQAQMKDC	1650
1651	MRELDD**TRAS**	**REEILAQAKE**	**NEKKLKSMEA**	**EMIQLQEELA**	**AAERAKRQAQ**	1700
1701	**QERDELADEI**	**ANSSGKGALA**	**LEE**KRRLEAR	IAQLEEE**LEE**	**EQGNTELIN**D	1750
1751	RLKKANLQ**ID**	**QINTDLNLER**	**SHAQKNENA**R	QQLERQNKEL	KVKLQEMEGT	1800
1801	**VKSKYK**ASIT	ALEAKIAQLE	EQ**LDNETKER**	**QAACKQVR**RT	EKKLKDVLLQ	1850
1851	VDDE**RRNAEQ**	**YKD**QADK**AST**	**R**LKQLKRQ**LE**	**EAEEEAQRAN**	**ASRRKLQREL**	1900
1901	**EDATETADAM**	**NREVSSLKNK**	**LRRGDLPFVV**	**PRRMARKGAG**	**DGSDEEVDGK**	1950
1951	**ADGAEAKPAE**					2000

B

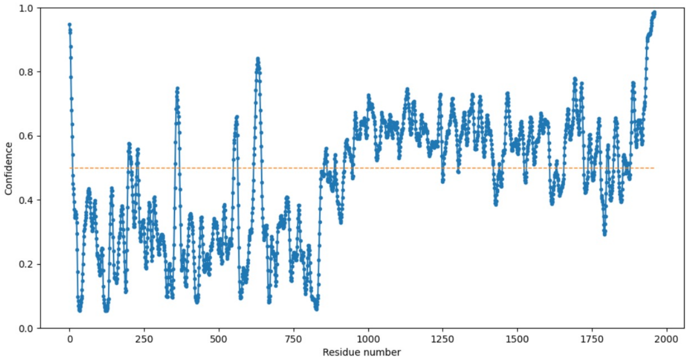

Supplementary Figure 7. Predicted disordered region of MYH9 protein

Disordered region of human MYH9 protein amino acids was predicted by protein disorder prediction system (PrDOS).
(A) All amino acids of the human MYH9 protein. Predicted disordered amino acids were red colored. (B) Schematic plots of the human MYH9 amino acids with the confidence of predicted disordered amino acids.
